# Relation between Exercise Training-Induced Changes in Oxygen Uptake Kinetics and the Power-Duration Relation

**DOI:** 10.1249/MSS.0000000000003991

**Published:** 2026-03-24

**Authors:** TZE-HUAN LEI, LUUK VOS, I-LIN WANG, SHUNSAKU KOGA, SIMON MARWOOD, BERNARD KORZENIEWSKI, RICHIE P. GOULDING

**Affiliations:** 1 College of Physical Education, Hubei Normal University, Huangshi, CHINA; 2 Department of Biomedical Engineering and Physics, Amsterdam UMC, Amsterdam, THE NETHERLANDS; 3 Applied Physiology Laboratory, Kobe Design University, Kobe, JAPAN; 4 School of Health & Sport Sciences, Liverpool Hope University, Liverpool, UNITED KINGDOM; 5 BioSimulation Center, Krakow, POLAND; 6 Department of Human Movement Sciences, Faculty of Behavioral and Movement Sciences, Vrije Universiteit Amsterdam, Amsterdam Movement Sciences, Amsterdam, THE NETHERLANDS

**Keywords:** COMPUTER MODEL, CRITICAL POWER, EXERCISE BIOENERGETICS, OXYGEN UPTAKE KINETICS, POWER-DURATION RELATION

## Abstract

**Purpose:**

This study aimed to determine the relation between training-induced changes in the variable values of the power-duration relation (critical power [CP] and $W^{\prime }$) and those of pulmonary oxygen uptake ($\dot{V}O_{2}$) kinetics (the fundamental phase time constant; $\;\tau_{{\dot{V}}_{O2}}$, and the slow component amplitude; $\dot{V}O_{2sc}$).

**Methods:**

Eleven healthy untrained males underwent 2 wk of severe-intensity exercise training. Before and after training, $\dot{V}O_{2max}$ was assessed via an incremental exercise test on a cycle ergometer, CP and $W^{\prime }$ were assessed via constant power determination trials, and $\dot{V}O_{2}$ kinetics were assessed during exercise at a power output 10% above CP (posttraining at the same absolute and relative intensity as pretraining). A previously described and validated computer model of the human skeletal muscle bioenergetic system was used to provide further insight into training-induced changes.

**Results:**

CP and $\tau_{{\dot{V}}_{O2}}$were strongly inversely correlated before and after training, and their training-induced changes were also correlated. Computer simulations suggested that increased oxidative phosphorylation activity (k_OX_) was the main factor determining the training-induced changes in CP and $\;\tau_{{\dot{V}}_{O2}}$. Exercise training increased $W^{\prime }$ and reduced the amplitude of the $\dot{V}O_{2sc}$, however, the training-induced changes in $W^{\prime }$ and $\dot{V}O_{2sc}$ were not correlated. Model simulations suggested that variations in k_OX_, the accessible phosphate (+creatine) pool (P_acc_), and the peak inorganic phosphate (P_i_) concentrations attained before task failure (Pi_peak_) could explain the observed training-induced alterations in $W^{\prime }$ and $\dot{V}O_{2sc}$.

**Conclusions:**

The present study suggests that the bioenergetic mechanisms underpinning CP and $\tau_{{\dot{V}}_{O2}}$are similar, whereas the relation between $W^{\prime }$ and $\dot{V}O_{2sc}$ appears somewhat more complex.

Tolerance to high-intensity exercise is well-described by the hyperbolic relation between external power and the tolerable duration of exercise (1–3). This relation is characterized by two parameters: critical power (CP), which represents the asymptote of the relation, and $W^{\prime }$, the curvature constant of the relation, which reflects a finite amount of work that can be performed above CP (4,5). CP represents the highest power output (PO) for which steady-state responses of oxygen consumption (i.e., pulmonary and muscle oxygen uptake, $\dot{V}O_{2}$), metabolite fluxes (e.g., intramuscular adenosine triphosphate [ATP] turnover), metabolite levels (e.g., high-energy phosphates, reduced nicotinamide adenine dinucleotide [NADH], and protons) and systemic and intramuscular acid-base status are attainable during exercise (6–8), and hence together with $W^{\prime }$, these parameters establish the tolerable duration of high-intensity exercise (9–11). Their physiological determinants have received considerable research attention, largely due to the fact that they set the limits of endurance performance and constrain physical capacity in aging and disease (9).

The fundamental response of pulmonary $\dot{V}O_{2}$ to constant load exercise is well-described by an exponential function, which is characterized by a time constant ($\tau_{{\dot{V}}_{O2}}$) that closely reflects the time constant of muscle $\dot{V}O_{2}$ kinetics (12–14). $\tau_{{\dot{V}}_{O2}}$is a sensitive indicator of aerobic function, being small (i.e., $\dot{V}O_{2}$ kinetics are fast) in trained athletes and large (i.e., $\dot{V}O_{2}$ kinetics are slow) in young sedentary individuals, elderly subjects, and diseased patients (9,15,16). CP has been shown to be strongly inversely correlated with $\tau_{{\dot{V}}_{O2}}$(9,15,17), and interventional studies have shown that experimental speeding of the $\dot{V}O_{2}$ kinetics is associated with an increase in CP (18–20), and experimental slowing of the $\dot{V}O_{2}$ kinetics is associated with a reduction in CP (21,22). This association has been interpreted to reflect the fact that both parameters share underpinning physiological and biochemical mechanisms. However, these mechanisms remain incompletely characterized.

Above the lactate threshold, the fundamental $\dot{V}O_{2}$ response is supplemented by the $\dot{V}O_{2}$ slow component ($\dot{V}O_{2sc}$), which elevates $\dot{V}O_{2}$ above that predicted from sub-lactate threshold exercise (23–25). This decline in work efficiency is thought to reflect the process of intramuscular fatigue (26), and hence the development of the $\dot{V}O_{2sc}$ is mechanistically linked to fatigue development during severe-intensity exercise (27–30). It has been suggested that task failure within the severe domain occurs due to the attainment of critical levels of peripheral fatigue (31,32), with the progression of the $\dot{V}O_{2sc}$ largely reflective of the development of the ongoing fatigue processes within the contracting muscle fibers (10,33). Since the $\dot{V}O_{2sc}$ is driven by fatigue and the loss of muscular efficiency, and the rate of fatigue development within the severe domain also determines $W^{\prime }$, it has been suggested that the $\dot{V}O_{2sc}$ and $W^{\prime }$ share underlying mechanisms (10,26). Indeed, a strong positive relation between the amplitude of the $\dot{V}O_{2sc}$ and $W^{\prime }$ has been reported (17), however, the mechanisms giving rise to these correlations have not been satisfactorily explained.

Recent *in silico* studies have provided insight into potential shared mechanisms supporting the $\tau_{{\dot{V}}_{O2}}$–CP and $\dot{V}O_{2sc}$–$W^{\prime }$ relations, via the postulation of a P_i_ double-threshold mechanism of muscle fatigue (34,35), which was preceded by the earlier fatigue-factor-F-based mechanism (35). This mechanism is based on three assumptions: 1) the $\dot{V}O_{2}$ and muscle metabolite slow components are underpinned by an additional ATP usage and this additional ATP usage occurs when P_i_ concentration exceeds a critical value (termed Pi_crit_); 2) exercise is terminated due to fatigue when P_i_ concentration reaches a peak value (Pi_peak_); and 3) a positive-feedback loop exists between P_i_ concentration and additional ATP usage, whereby increases in P_i_ due to additional ATP usage stimulates further increases in the additional ATP usage, which in turn drives further increases in P_i_.

The P_i_ double-threshold has received support from recent experimental studies (36), and the model simulations based on the assumptions highlighted above result in the appearance of several emergent phenomena, such as the heavy-intensity domain (37) and the hyperbolic relation between power and the duration of exercise in the severe-intensity domain (33–35). Model simulations further demonstrate that alterations in kinetic properties of the system such as oxidative phosphorylation (OXPHOS) activity, each-step activation (ESA) intensity, Pi_crit_, Pi_peak_, the accessible phosphate (+creatine) pool (P_acc_), and intramyocellular O_2_ pressure affect $\tau_{{\dot{V}}_{O2}}$ and CP in opposite directions (34,35,38,39), consistent with experimental data (18–22,40). On the other hand, model simulations suggested that the main factor explaining the positive linear relation between the amplitude of the $\dot{V}O_{2sc}$ and $W^{\prime }$ in different individuals (17) was the rate constant of additional ATP usage (k_add_) (39).

The aim of the present study was to examine the shared mechanisms sustaining the $\dot{V}O_{2}$ kinetics and the parameters of the power-duration relation using a combined experimental and theoretical approach. We determined $\dot{V}O_{2}$ kinetics and the power-duration relation both before and after a short-term (2 wk), high-intensity exercise training intervention. We hypothesized that 1) CP would be inversely related to $\tau_{{\dot{V}}_{O2}}$ both before and after training at the same absolute power output, and 2) that training-induced changes in CP would be correlated with changes in $\tau_{{\dot{V}}_{O2}}$determined at the same absolute power output. Utilizing a previously validated model of the human skeletal muscle bioenergetic system (35), we also hypothesized that 3) alterations in $\tau_{{\dot{V}}_{O2}}$(denoted *t*_0.63_ in simulations), CP, and their relation posttraining would be largely explained by training-induced increases in OXPHOS activity (k_OX_).

Endurance exercise training typically leads to either a decrease or no change in $W^{\prime }$ and a reduction in the $\dot{V}O_{2sc}$ amplitude at a given absolute power output (41–43), consistent with prior simulation studies (34). However, accurate quantification of the $\dot{V}O_{2sc}$ is complicated by the fact that its magnitude depends on the duration of exercise. Exercise training would likely extend the tolerable duration of exercise at the same absolute power output as the preintervention criterion trial, altering the contribution of the $\dot{V}O_{2sc}$ to the end-exercise $\dot{V}O_{2}$. Hence, to accurately describe the $W^{\prime }$–$\dot{V}O_{2sc}$ relation, we also conducted comparisons at the same relative intensity (i.e., 10% above CP) pre- versus postintervention. Based on prior observations of a positive $\dot{V}O_{2sc}$–$W^{\prime }$ association, we therefore hypothesized 4) that $W^{\prime }$ would be positively related to the amplitude of the $\dot{V}O_{2sc}$ both before and after training at the same relative exercise intensity, and 5) that changes in $W^{\prime }$ would be correlated with changes in the $\dot{V}O_{2sc}$. Utilizing the model described above, we also expected that 6) the $\dot{V}O_{2sc}$–$W^{\prime }$ relation would be explained by variation in biochemical factors such as k_OX_, k_add_, P_acc_, and/or Pi_peak_.

## METHODS

### Participants

Eleven healthy, recreationally active but untrained Asian men were recruited for this study (participant characteristics reported in Table 1). This study was approved by the University Joint Institute Review Board (JLSU-IRB2023003) and performed in accordance with the latest version of the Declaration of Helsinki and registered with the China Clinical database (ChiCTR2300071860). Each participant provided verbal and written informed consent before participation.

**TABLE 1.** Cohort characteristics (values displayed are mean ± SD).

Characteristics	Pretraining	Posttraining	*P* Value
Age (yr)	25 ± 4	-	-
Height (cm)	178 ± 5	-	-
Body mass (kg)	77.4 ± 8.5	77.3 ± 8.5	0.34
BMI (kg·m^−2^)	24.3 ± 2.1	24.3 ± 2.1	0.34
$\dot{V}O_{2}$ (L·min^−1^)	3.10 ± 0.56	3.60 ± 0.30*	0.001
$\dot{V}O_{2}$ (mL·kg^−1^·min^−1^)	40.7 ± 7.8	46.4 ± 5.3*	0.002
Peak power (W)	264 ± 30	312 ± 19*	<0.0001
GET (L·min^−1^)	1.84 ± 0.25	2.20 ± 0.18*	<0.0001
GET (mL·kg^−1^·min^−1^)	24.0 ± 3.97	28.3 ± 3.0*	<0.0001
GET (W)	119 ± 18	158 ± 15*	<0.0001
GET (%$\dot{V}O_{2max}$)	59.6 ± 6.2	61.3 ± 3.1	0.32
MRT (s)	42.9 ± 21.3	40.3 ± 23.6	0.75
Training power output (W)	172 ± 22	-	-

* Significant difference from pre- to posttraining (*P* < 0.05). Means were analyzed using paired samples *t* tests.

BMI, body mass index; $\dot{V}O_{2max}$, maximal oxygen uptake; MRT, mean response time.

### Experimental Overview

All participants reported to a temperature-controlled laboratory (24.5°C ± 0.5°C, 50.5% ± 5% relative humidity) to undergo the following procedures both before and after a 2-wk exercise training intervention: 1) a maximal ramp incremental exercise test, 2) five CP determination trials, and 3) two trials performed at a power output situated 10% above CP for determination of the $\dot{V}O_{2}$ kinetics. Postintervention, two trials were performed at a power output situated 10% above the preintervention CP (i.e., at the same absolute power output as preintervention), and a further two trials were performed at a power output situated 10% above the postintervention CP (i.e., the same relative power output). These postintervention trials for determination of the $\dot{V}O_{2}$ kinetics were performed in a randomized order. In between the pre- and posttraining assessments, participants completed a 2-wk high-intensity exercise training period consisting of seven sessions per week (i.e., daily training) on consecutive days at a power output situated 10% above CP for 15 min. The actual power output utilized for the training study was adjusted for each individual to ensure that participants were close to the limit of tolerance at the end of each session, but that each session could last for the full 15 min. Each experimental trial was separated by at least 48 h to ensure adequate recovery. Each experimental trial was conducted at the same time of day to eliminate the effect of diurnal rhythm on blood pressure and body temperature fluctuations, and was performed 2 h postprandial. Furthermore, participants abstained from alcohol/caffeine intake and avoided high-sodium foods 48 h before all experimental trials. Height and body mass were measured at the first visit using a stadiometer (Seca, Hamburg, Germany; accurate to 0.1 cm) and scale (Jadever, Taipei, Taiwan; accurate to 10 g).

### Incremental Testing and Constant Power Trials

All participants completed a maximal ramp incremental exercise test on a cycle ergometer (Ergoselect 100, Ergoline GmbH, Bitz, Germany) for determination of $\dot{V}CO_{2}$, the gas exchange threshold (GET), and the power outputs for subsequent visits. Each test consisted of a 4-min baseline period of cycling at 20 W, followed by a ramped, linear increase in work rate of 20 W·min^−1^ at a fixed cadence of 70 rpm until the participant could no longer maintain the cadence above 60 rpm despite strong verbal encouragement. Ventilatory and gas exchange variables were measured continuously breath-by-breath throughout each test. $\dot{V}O_{2}$ was confirmed by either 1) the occurrence of a plateau in $\dot{V}E$ despite an increasing workload during the ramp incremental test or 2) the occurrence of a plateau in the plot of $\dot{V}O_{2}$ versus power output when determined from the discontinuous tests used to determine CP, as described by Day et al. (44). The GET was independently verified by two different investigators (T.-H. L. and R. P. G.) using the following criteria: 1) a disproportionate increase in CO_2_ production ($\dot{V}E$) relative to $\dot{V}CO_{2}$; 2) an increase in the ventilatory equivalent for O_2_ (i.e., $\dot{V}O_{2}$/$\dot{V}O_{2max}$) without an increase in the ventilatory equivalent for CO_2_ (i.e., $\dot{V}O_{2max}$/$\dot{V}O_{2}$); and an increase in end tidal O_2_ tension with no change in end tidal CO_2_ tension. All power outputs utilized in subsequent tests were corrected by the $\dot{V}O_{2}$ mean response time, which was determined as described previously (18,19,40).

The power-duration relation was determined by five constant power output trials that were selected to elicit a range of times to task failure spanning 2–15 min. These power outputs were initially selected based on performance in the incremental test and calculated to be in the range of 50%Δ (i.e., halfway between GET and $\dot{V}O_{2max}$) to 110% $\dot{V}O_{2}$. All tests began with a 4 min period of baseline pedaling at 20 W, before an abrupt transition to the desired power output was applied and participants exercised until task failure. Time to task failure was recorded to the nearest second. If the peak $\dot{V}O_{2}$ attained at the end of any given trial was less than 95% of that attained in the incremental test, that trial was repeated. Conversely, if the peak $\dot{V}O_{2}$ attained at the end of any given trial was greater than 105% of that attained in the incremental test, the incremental test was repeated. In all cases, $\dot{V}CO_{2}$ was verified. Subsequent to the power-duration relation determination trials, a further two exhaustive trials were performed at 10% above CP on separate days in the same manner, to enable determination of the $\dot{V}E$ kinetics. Postintervention, these trials were performed on four rather than two occasions, with two performed at the same absolute power output as pretraining, and two performed at the same relative power output as pretraining. These trials served to reduce the width of the confidence intervals associated with the modeling of the $W^{\prime }$ kinetics.

### Gas Exchange and Ventilation Measurements

Expired respiratory gases were collected from a mixing chamber-based metabolic system with breath-resolved flow measurement and continuous gas sampling and analyzed online for $T=W^{\prime }/\left(P-CP\right)$, $W=CP\times T+W^{\prime }$ and $P=W^{\prime }\times \left({\;}^{1}/{\;}_{T}\right)+CP$ (Max II, AEI, Automated Engineering Inc. [AEI Technologies], Bastrop, TX, USA). Inspired and expired O_2_ and CO_2_ concentrations were measured using paramagnetic and infrared gas analyzers, respectively, whereas volume measurements were recorded via a pneumotach. Gas exchange variables were computed online and subsequently time-averaged using a 10 s average during the ramp incremental test and every 3 s during the subsequent trials. This system was calibrated before each trial using a zero and *β*-standard gas concentrations (5% CO_2_, 21% O_2_, balance N_2_) and a 3-L syringe (Hans Rudolph 3L Calibration Syringe, Shawnee, KS, USA), according to the manufacturer’s instructions.

### Data Analysis

CP and $W^{\prime }$ were estimated using the three following models: the hyperbolic power-time model, where time to task failure is plotted against power output (equation 1); the linear work-time model, where time is plotted against work done (equation 2); and the linear inverse of time model, where power is plotted against the inverse of time (equation 3), as follows:


$$T=W^{\prime }/\left(P-CP\right)$$ (1)

$$W=CP\times T+W^{\prime }$$ (2)

$$P=W^{\prime }\times \left({\;}^{1}/{\;}_{T}\right)+CP$$ (3)

Where P is power, T is time, W is work done, CP is critical power, and $W^{\prime }$ is the finite work capacity available above CP. The standard error of the parameter estimates for CP and $\dot{V}O_{2}$ were expressed as a coefficient of variation relative to the parameter estimate (CV%). The model with the lowest average CV% was then used for all subsequent analyses (i.e., the best individual fit method (45)).

$\dot{V}O_{2}$ data were edited to remove aberrant values (i.e., >4 standard deviation [SD] outside the local 5-breath mean). The edited $\dot{V}O_{2}$ data from the constant power exercise tests were then linearly interpolated (1 s), and identical bouts were time-aligned and ensemble-averaged across each transition for each subject. The first 20 s of data were considered reflective of the cardiodynamic phase and were thus discarded. A monoexponential model with time delay was then fitted to the data, as follows:

1. $\dot{V}O_{2}$ (*t*) = $A_{{\dot{V}}_{O2}}$_b_ + $TD_{{\dot{V}}_{O2}}$× (1 – e ^– (*t* –^$\tau_{{\dot{V}}_{O2}}$^)^)/$\dot{V}O_{2}$)

Where $\dot{V}O_{2}$ (*t*) is the value of $\dot{V}O_{2}$ at any time *t*, $A_{{\dot{V}}_{O2}}$_b_ is the baseline value measured over the final 30 s of baseline cycling, $\dot{V}O_{2}$is the amplitude of increase in $TD_{{\dot{V}}_{O2}}$ above baseline, $\tau_{{\dot{V}}_{O2}}$ is the time delay, and $\dot{V}O_{2sc}$ is the time constant of the response. The model was constrained to exclude the slow component and hence isolate the fundamental phase. The onset of the $\dot{V}O_{2}$ was identified using purpose-designed programming in MATLAB (MathWorks, Natick, MA, USA), which iteratively fits a monoexponential function to the $\tau_{{\dot{V}}_{O2}}$ data, starting at 60 s until the window encompasses the entire response. The estimated $\tau_{{\dot{V}}_{O2}}$ values for each fitting window are then plotted against time, with the onset of the slow component determined as the point at which $\tau_{{\dot{V}}_{O2}}$, the reduced chi squared value and the 95% confidence interval for $\dot{V}O_{2sc}$consistently deviated from a previously “flat” profile. The amplitude of the $\dot{V}O_{2}$ was determined by calculating the difference between $\dot{V}O_{2}$ at 600 s (i.e., mean 30 s $\dot{V}O_{2}$ over centered on 600 s, the shortest duration at which any individual reached the limit of tolerance pretraining) and $A_{{\dot{V}}_{O2}}$_(b)_ + $\dot{V}O_{2sc}$. The amplitude of the $\dot{V}O_{2sc}$ was also calculated at the end of exercise, but this did not change any outcome and hence these results are not presented here. Given the duration and intensity-dependence of the $W^{\prime }$, correlations with $\tau_{{\dot{V}}_{O2}}$ were performed at both the same absolute and relative power outputs. In contrast, $\dot{V}O_{2}$comparisons were presented at the same absolute rather than relative power output, as this comparison involves one fewer parameter change, namely ATP usage activity, and therefore allows a more direct mechanistic interpretation.

### Statistical Analysis

All statistical analyses were performed with GraphPad Prism (Prism version 7.00, GraphPad Software, San Diego, CA, USA). The normality of the data was examined by the Kolmogorov–Smirnov Test. All data were normally distributed and therefore, means for outcome parameters from pre- to postintervention were compared using paired samples *t* tests. Correlations were explored between variables of interest using the Pearson correlation coefficient. Data presented are means ± SD, unless stated otherwise. Statistical significance was accepted at *P* < 0.05.

### Computer Simulations

A dynamic computer model of the skeletal muscle bioenergetic system, including a detailed model of OXPHOS, was used for theoretical studies (33,46). The model involves the ESA mechanism, whereby ATP usage and all ATP supply processes taken into account explicitly within the model (including all OXPHOS complexes, NADH supply, and glycolysis) are simultaneously activated in parallel at the onset of work by some cellular factor/mechanism (likely involving cytosolic and mitochondrial Ca^2+^ ions and protein (de)phosphorylation) (46,47). The model also involves the P_i_ double-threshold mechanism of muscle fatigue described above (35).

Within the computer model, the rate constants that appear in kinetic equations for all OXPHOS complexes (complex I, complex III, complex IV, ATP synthase, ATP/adenosine diphosphate [ADP] carrier, and P_i_ carrier) and the NADH supply block (k_C1_, k_C3_, k_C4_, k_SN_, k_EX_, k_PI_, and k_DH_, respectively) can be grouped into a single rate constant of OXPHOS: k_OX_, which corresponds to OXPHOS activity. In the standard model version, corresponding to young healthy untrained physically active individuals, the relative k_OX_ is scaled to 1. This is a mean value, which can of course vary markedly between individuals. The complete model description is given in (33) (with the exception of the complete P_i_ double-threshold mechanism, which is described by (35)) and is also located on the website: http://bernardkorzeniewski1.pl.

In computer simulations of the time courses of $\dot{V}O_{2}$ during the rest → constant-work transitions, the ATP usage activity (A_UT_, scaled to 1 at rest) was increased instantaneously from 1 to a desired value: 78.2 (comparisons were performed pretraining and posttraining at the same A_UT_, analogous to the comparisons performed at the same absolute power output pre- and posttraining in the experimental portion of the study) or 89.3 (posttraining only, analogous to the comparisons performed at the same relative intensity pre- and posttraining in the experimental portion of the study). Additionally, several different values of A_UT_ were utilized for the determination of the power-duration curves. The posttraining A_UT_ value of 89.3 was selected to terminate exercise because of fatigue after about 15 min; which is close to the mean tolerable duration of exercise performed at 10% above CP posttraining. The pretraining A_UT_ value of 78.2 was calculated by dividing 89.3 by 1.14, as the posttraining CP = 177 W was 1.14 times higher than pretraining CP = 155 W. For this reason, the A_UT_ of 89.3 in the trained state was considered to be reflective of the same relative intensity as an A_UT_ of 78.3 in the untrained state. In whole-body exercise, 1 A_UT_ unit corresponds to a power output of about 3 W (2–4 W, depending e.g., on working muscle mass and exercise type). The simulations continued for 50 min or until the moment when P_i_ reached Pi_peak_, and exercise was terminated because of fatigue.

The power-duration (critical ATP usage activity A_UTcrit_−*t*_end_) dependence was simulated through recording the times at which exercise was terminated because of fatigue at different ATP usage activities (A_UTs_), which are analogous to different power outputs.

Concerning the $W^{\prime }$ on-kinetics and power-duration dependence, four groups of simulations were carried out: one for pretraining and three posttraining. In the posttraining simulations, different parameter values were changed in relation to pretraining. The parameters involved were OXPHOS activity (k_OX_), the accessible phosphate (+creatine) pool (PCr + Cr + P_i_) (P_acc_) (38), and peak P_i_ (Pi_peak_) at which exercise is terminated because of fatigue. These were the only combinations of parameters appearing within the model that gave, at least semi-quantitatively, the expected results, especially the training-induced $\tau_{{\dot{V}}_{O2}}$ increase (discussed later). The sets of pre- and posttraining parameter values (k_OX_, OXPHOS activity; P_acc_, accessible phosphate (+creatine); Pi_peak_; peak P_i_) are enumerated below. These values were adjusted to obtain relative changes in variable values comparable to those encountered in the experiment.

untrained: k_OX_ = 1.0, P_acc_ × 1.0, Pi_peak_ = 25 mM;

trained 1: k_OX_ = 1.14, P_acc_ × 1.0, Pi_peak_ = 25 mM;

trained 2: k_OX_ = 1.14, P_acc_ × 0.98, Pi_peak_ = 25 mM;

trained 3: k_OX_ = 1.14, P_acc_ × 1.0, Pi_peak_ = 25.5 mM,

where ×1.0 or ×0.98 means multiplicity of the “standard” value. The value of k_OX_ × 1.14 was chosen to match the experimental increase in CP. Values of P_acc_ × 0.98 and Pi_peak_ = 25.5 mM were chosen to explore whether modest, physiologically plausible changes in P_acc_ and Pi_peak_, in combination with the experimentally constrained increase in k_OX_, could qualitatively reproduce the relative magnitude and direction of the observed training effects on $\dot{V}O_{2sc}$, the $W^{\prime }$, CP, and $\dot{V}O_{2max}$. To assess model sensitivity, a range of parameter combinations was explored; the cases presented are illustrative examples chosen to demonstrate how specific, physiologically plausible parameter changes influence model behavior, rather than to provide an exhaustive parameter sweep.

## RESULTS

The 2-wk high-intensity endurance training intervention resulted in robust improvements in $\dot{V}O_{2}$, the peak power output attained during incremental exercise, the $W^{\prime }$ at the GET, and its associated power output (Table 1). The exercise training sessions were performed at 172 ± 22 W, which was greater than the preintervention CP (155 ± 19 W, *P* = 0.0002).

### Effects of Training on the Power-Duration Relation

**FIGURE 1:**
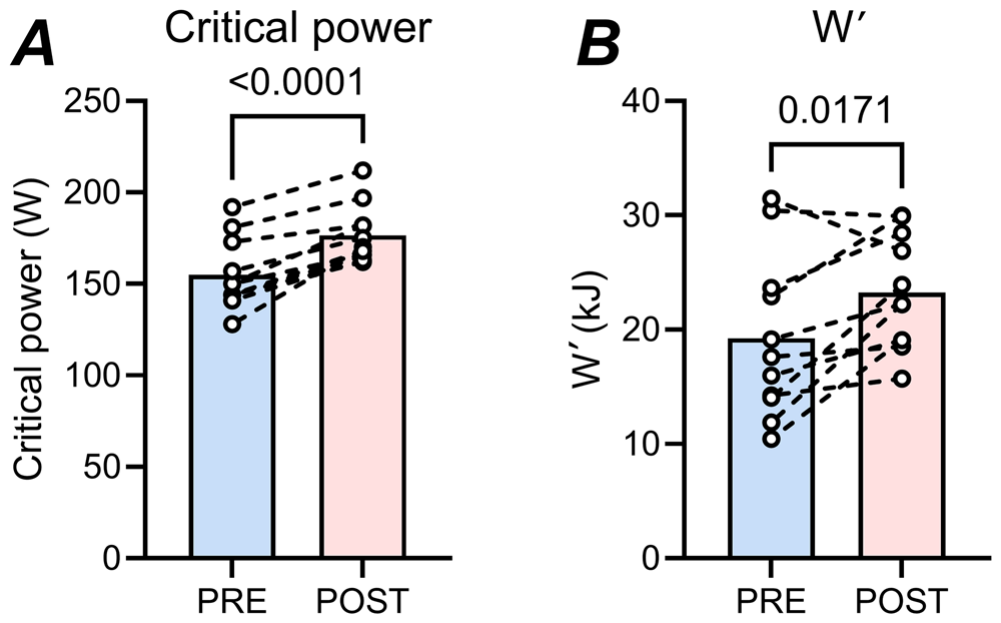
Effects of training on the power-duration relation. A, Group mean (*bars*) and individual changes (*circles* and *dashed lines*) in critical power from pre- to postintervention. B, Group mean (*bars*) and individual changes (*circles* and *dashed lines*) in $\tau_{{\dot{V}}_{O2}}$ from pre- to postintervention. *P* values of the paired samples *t* tests are displayed above each panel.

The group mean ± SD and individual changes in CP and $W^{\prime }$ from pre- to postintervention are shown in Fig. 1A, B, respectively. Both CP (PRE: 155 ± 19 W, CV: 1.9% ± 1.0%; POST: 177 ± 16 W, CV: 1.9% ± 0.9%; *P* < 0.0001) and $\dot{V}O_{2}$ (PRE: 19.2 ± 7.0 kJ, CV: 9.6% ± 6.6%; POST: 23.3 ± 5.0 kJ, CV: 6.6% ± 3.4%; *P* = 0.017) were increased from pre- to postintervention (Fig. 1).

### Effects of Training on Pulmonary Oxygen Uptake Kinetics

**FIGURE 2:**
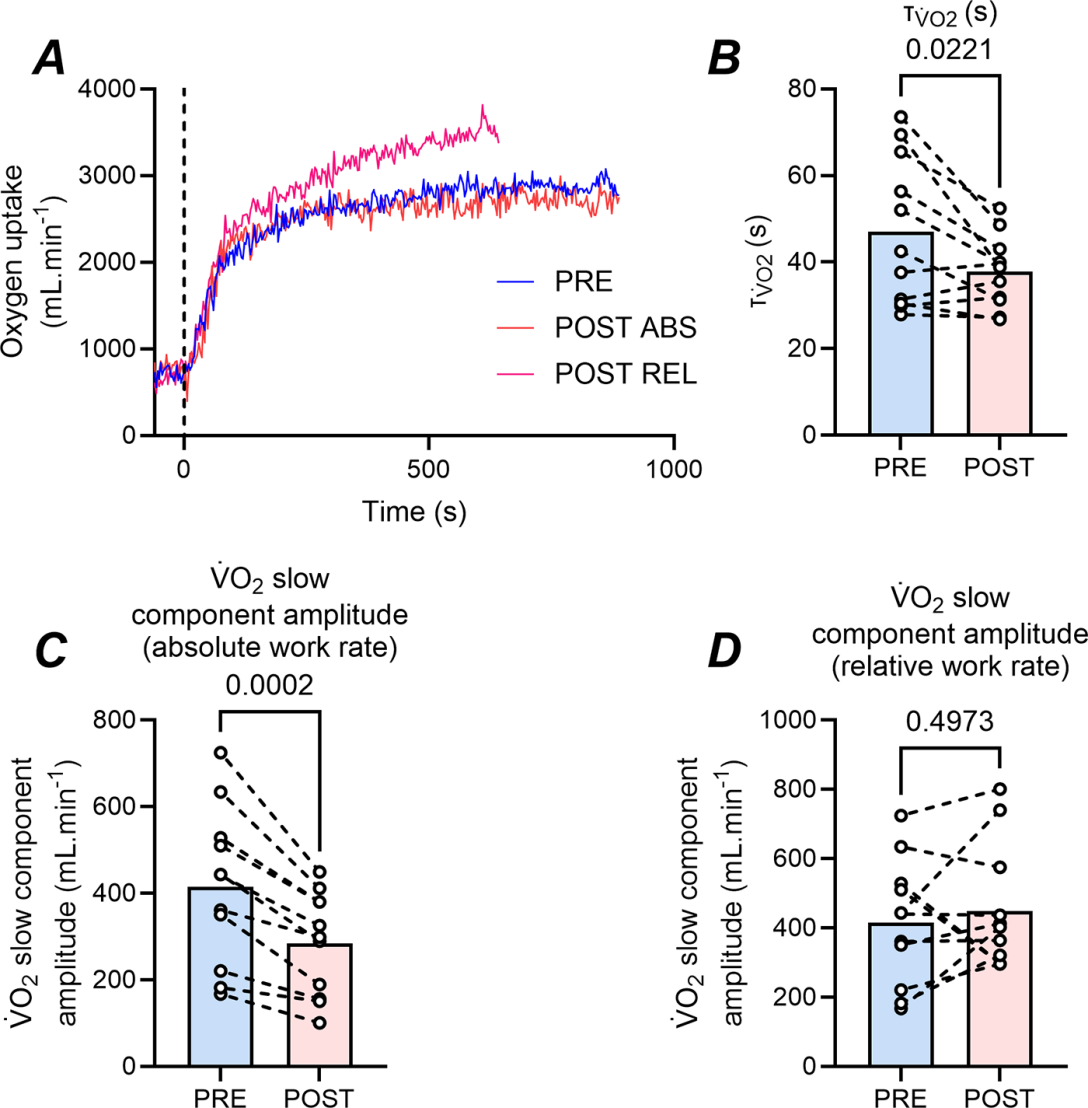
Effects of training on pulmonary oxygen uptake kinetics. A, Representative example of pulmonary oxygen uptake ($\tau_{{\dot{V}}_{O2}}$) responses to severe-intensity exercise performed at a power output situated 10% above the preintervention critical power (PRE) and the same absolute (POST ABS) and relative (POST REL) power output postintervention. B, Group mean (*bars*) and individual changes (*circles* and *dashed lines*) in the time constant of the fundamental phase of $\dot{V}O_{2sc}$ kinetics ($W^{\prime }$) from pre- to postintervention. C, Group mean (*bars*) and individual changes (*circles* and *dashed lines*) in the amplitude of the $W^{\prime }$ slow component ($\dot{V}O_{2}$) from pre- to postintervention determined at the same absolute power output. D, Group mean (*bars*) and individual changes (*circles* and *dashed lines*) in the amplitude of the $\dot{V}O_{2}$ slow component ($\dot{V}O_{2sc}$) from pre- to postintervention determined at the same relative power output. *P* values for each paired samples *t* test are displayed above each panel.

The pulmonary $\dot{V}O_{2sc}$ responses to exercise performed 10% above CP in a representative participant before and after training at the same absolute power output are displayed in Figure 2A. $\dot{V}O_{2sc}$was reduced at post- compared with pretraining at the same absolute power output (PRE: 47 ± 17 s, POST: 38 ± 8 s, *P* = 0.022, Fig. 2B). Similarly, the amplitude of the $\tau_{{\dot{V}}_{O2}}$ (PRE: 0.42 ± 0.18 L·min^−1^, POST: 0.28 ± 0.12 L·min^−1^, *P* = 0.0002, Fig. 2C) was blunted posttraining at the same absolute power output. However, the $\tau_{{\dot{V}}_{O2}}$ was not altered posttraining at the same relative power output (amplitude: 0.45 ± 0.18 L·min^−1^, Fig. 2D, *P* = 0.50).

### The Relation between Parameters of the Power-Duration Relation and Pulmonary Oxygen Uptake Kinetics

**FIGURE 3:**
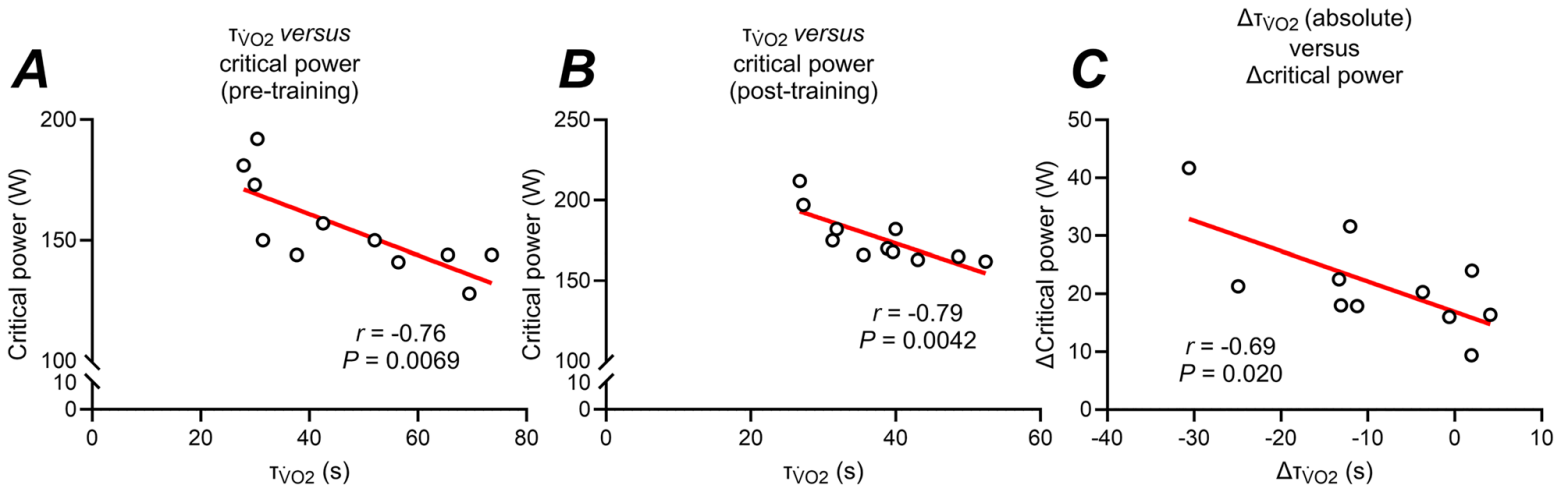
Relation between the fundamental phase oxygen uptake time constant ($\dot{V}O_{2max}$) and critical power (CP). The relation between the pretraining $\dot{V}O_{2sc}$_O2_ and CP is shown in (A), whereas the relation between the posttraining $\dot{V}O_{2sc}$ determined at the same absolute power output as pretraining with CP is shown in (B). C, Relation between the training-induced change in $W^{\prime }$ (Δ$W^{\prime }$) and CP (ΔCP). *Clear circles* reflect individual data points, whereas the *solid red line* displays the line of best fit from the Pearson correlation. *r* and *P* values are displayed in the inset of each panel.

$\dot{V}O_{2}$was inversely correlated with CP both before (Fig. 3A, *r* = −0.76, *P* = 0.0069) and after training (Fig. 3B, *r* = −0.79, *P* = 0.0042). The training-induced change in $\dot{V}O_{2sc}$ (i.e., Δ$\dot{V}O_{2sc}$) was inversely correlated with the training-induced change in CP (i.e., ΔCP) (Fig. 3C, *r* = −0.69, *P* = 0.020).

**FIGURE 4:**
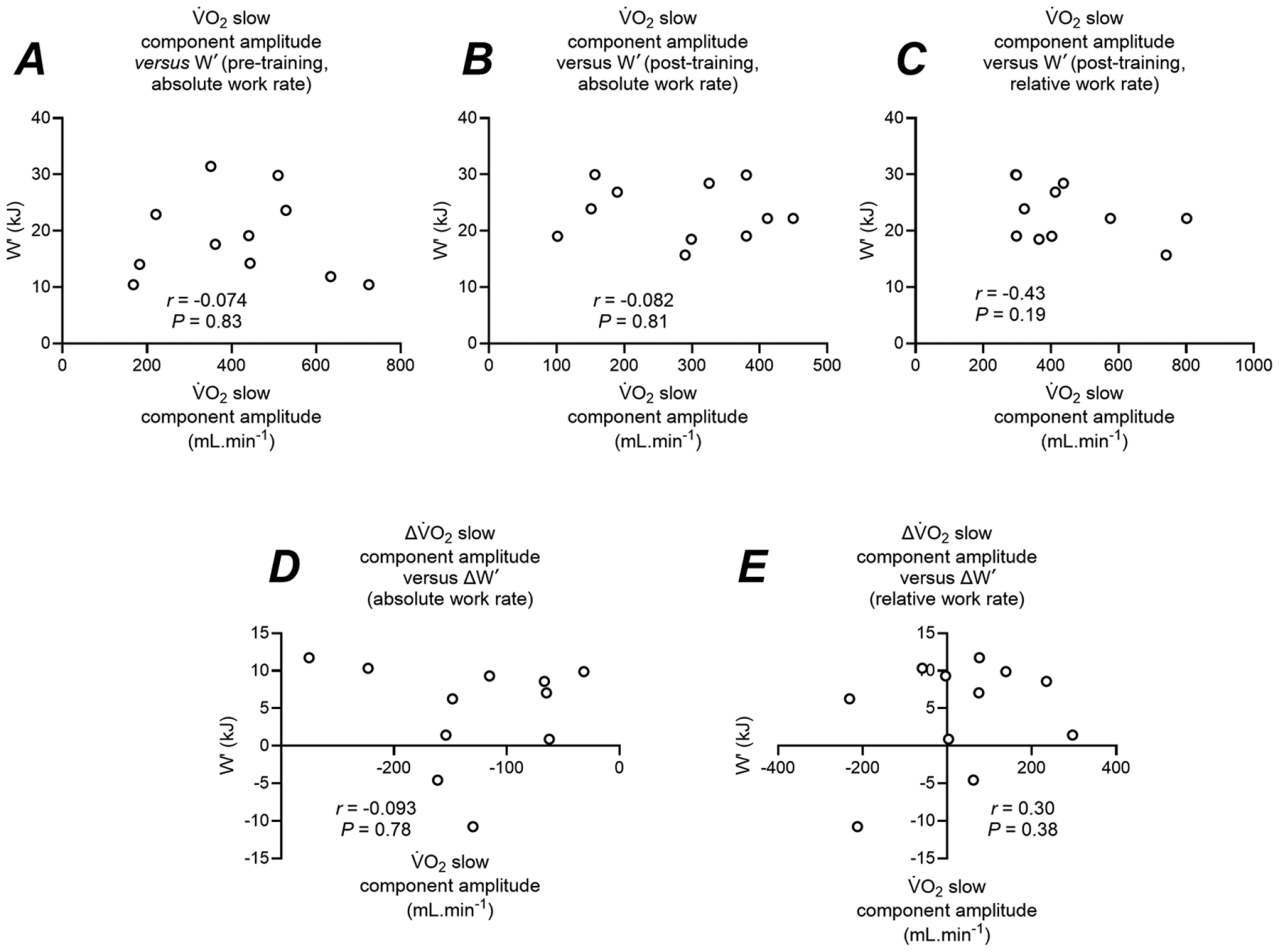
Relation between the slow component of oxygen uptake ($\tau_{{\dot{V}}_{O2}}$) kinetics and $\tau_{{\dot{V}}_{O2}}$. The relation between the amplitude of the $W^{\prime }$ slow component and $\dot{V}O_{2sc}$ is shown at pretraining (A) and posttraining at the same absolute power output as pretraining (B). C, The posttraining relation between the amplitude of the $W^{\prime }$ slow component and $\dot{V}O_{2sc}$ at the same relative power output as pretraining. D, The relation between training-induced changes in the amplitude of the $\dot{V}O_{2sc}$ slow component and $W^{\prime }$ when determined at the same absolute power output as pretraining, whereas (E) shows the same relation determined at the same relative power output as pretraining. *Clear circles* reflect individual data points. *r* and *P* values are displayed in the inset of each panel. No relation was significant.

The absolute amplitude of the $W^{\prime }$ was not related to $W^{\prime }$ either at pretraining (*r* = −0.074, *P* = 0.83, Fig. 4A) or posttraining at the same absolute (Fig. 4B, *r* = −0.082, *P* = 0.81) or relative intensity (Fig. 4C, *r* = −0.43, *P* = 0.19). Moreover, the training-induced change in the slow component amplitude, measured posttraining at either the same absolute (Fig. 4D, *r* = −0.093, *P* = 0.78) or relative intensity (Fig. 4E, *r* = 0.30, *P* = 0.38), was not correlated with the training-induced change in $\tau_{{\dot{V}}_{O2}}$.

### Simulated Pretraining and Posttraining $\dot{V}O_{2sc}$ On-Kinetics for the Same Absolute and Relative Power Output (PO)

**FIGURE 5:**
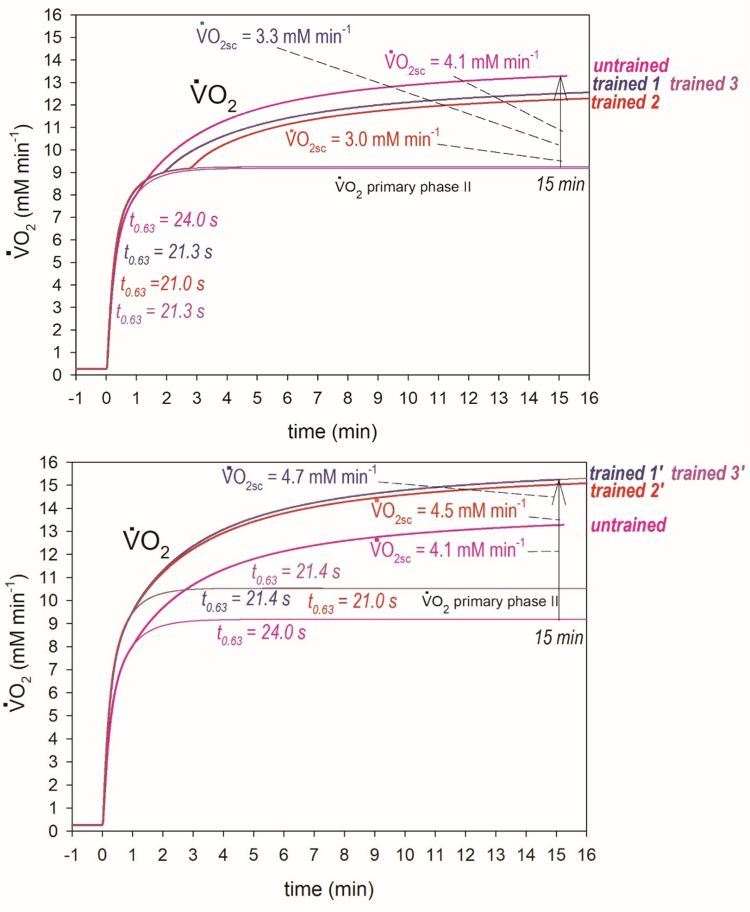
Simulated muscle oxygen uptake ($\dot{V}O_{2sc}$) on-kinetics in the untrained state and three different scenarios reflecting the trained state (trained 1–3, respectively) at the same absolute (upper panel) and relative (lower panel) ATP usage activity (A_UT_). Upper panel: All simulations were performed at the same absolute work intensity as in the untrained state (ATP usage activity A_UT_ = 78.2). In trained 1, the rate constant describing overall oxidative phosphorylation (OXPHOS) activity (k_OX_) was increased to 1.14 times the untrained value. In trained 2, in addition to the training-induced increase in k_OX_, the accessible phosphate pool (P_acc_) was decreased to 0.98 times the untrained value. In trained 3, in addition to the training-induced increase in k_OX_, the peak P_i_ value at which exercise was terminated (Pi_peak_) was increased from 25.0 mM (untrained) to 25.5 mM (see Table 1). The values of the transition time of the primary phase II and the magnitude of the slow component (after 15 min of exercise) of the $W^{\prime }$ on-kinetics are labeled on the figure. The time courses for “trained 1” and “trained 3” overlap. Lower panel: The simulation for the untrained state (trained) was performed at the work intensity characteristic for this state (ATP usage activity A_UT_ = 78.2). All simulations for the trained state (trained 1–3) were performed at the same relative work intensity as in the untrained state (ATP usage activity A_UT_ = 89.3). The time courses for “trained 1” and “trained 3” overlap until ~15 min of exercise; for “trained 3” exercise continues until 37.4 min.

Simulations of the $W^{\prime }$ on-kinetics in different posttraining scenarios (i.e., with different parameter changes) for the same absolute A_UT_ resulted in a decrease in *t*_0.63_ in relation to pretraining: from 24.0 s (untrained) to 21.3 s (trained 1 and trained 3, increase in k_OX_ or simultaneous increase in k_OX_ and increase in Pi_peak_, respectively) or 21.0 s (trained 2, increase in k_OX_ and decrease in P_acc_) (Fig. 5). The reduction in *t*_0.63_ was largely due to the training-induced increase in k_OX_. The $\dot{V}O_{2sc}$ amplitude after 15 min of exercise was decreased as a result of training: from 4.1 mM·min^−1^ in the untrained state to 3.3 mM·min^−1^ (trained 1 and trained 3) or 3.0 mM·min^−1^ (trained 2) in the trained state. In the untrained state, exercise was terminated because of exhaustion after 15.2 min. In all three trained cases, exercise at the same A_UT_ was not terminated before 50 min due to fatigue. Exercise at the same A_UT_ was thus shifted from the severe to the heavy exercise intensity domain as a result of training.

Simulations of the $\tau_{{\dot{V}}_{O2}}$ on-kinetics in different posttraining scenarios (i.e., with different parameter changes) for the same relative work intensity resulted in similar decreases in *t*_0.63_ in relation to pretraining as for simulations performed at the same absolute work intensity: from 24.0 s (untrained) to 21.4 s (trained 1 and trained 3) or 21.0 s (trained 2) (Fig 5). However, the amplitude of the $\tau_{{\dot{V}}_{O2}}$ at the same relative intensity was moderately increased as a result of training: from 4.1 mM·min^−1^ (untrained) to 4.7 mM·min^−1^ (trained 1 and trained 3) or 4.5 mM·min^−1^ (trained 2). In the untrained state, the tolerable duration of exercise was 15.2 min (severe exercise intensity domain), whereas in trained 1, the tolerable duration of exercise was 15.1 min (severe exercise intensity domain). In trained 2, the simulations were not terminated due to fatigue, whereas in trained 3, the tolerable duration of exercise was 37.4 min.

**FIGURE 6:**
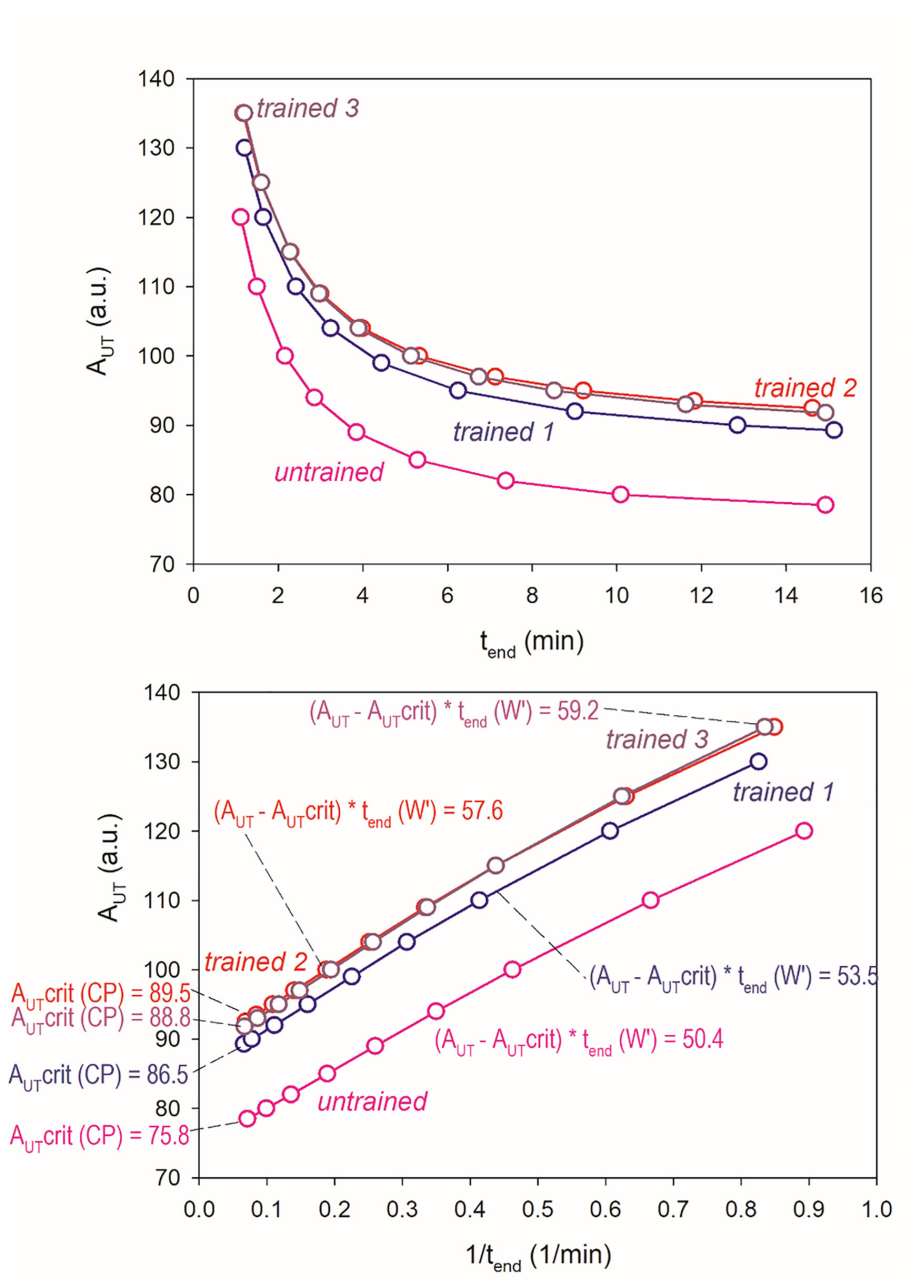
Simulated A_UT_-*t*_end_ (power-duration) and A_UT_−1/*t*_end_ (power−1/duration) relation in the untrained state and three different scenarios reflecting the trained state with different alterations in parameter values (see Table 2). In trained 1, the rate constant describing overall oxidative phosphorylation (OXPHOS) activity (k_OX_) was increased to 1.14 times the untrained value. In trained 2, in addition to the training-induced increase in k_OX_, the accessible phosphate pool (P_acc_) was decreased to 0.98 times the untrained value. In trained 3, in addition to the training-induced increase in k_OX_, the peak P_i_ value at which exercise was terminated (Pi_peak_) was increased from 25.0 mM (untrained) to 25.5 mM (see Table 2). The values of the critical ATP usage activity (A_UTcrit_), analogous to critical power (CP), and the work done above CP ([A_UT_−A_UTcrit_] × *t*_end_), analogous to the curvature constant ($\dot{V}O_{2}$), are shown.

**TABLE 2.** Relative training-induced changes in selected variable values in experimental data (exp.) and three groups of computer simulations for posttraining (trained 1, trained 2, and trained 3).

Intervention:	Exp.	Trained 1	Trained 2	Trained 3
Variable				
$\dot{V}O_{2}$	+16%	+15%	+19%	+19%
$\tau_{{\dot{V}}_{O2}}$ (the same absolute work intensity [A_UT_])	−38 %	−20%	−27%	−20%
$\tau_{{\dot{V}}_{O2}}$ (the same relative work intensity [A_UT_])	no st.-sign.	+15%	+10%	+15%
	(+10%–15%)			
*t*0.63	−19%	−11%	−13%	−11%
CP	+14%	+14%	+19%	+18%
W′	+21%	+6%	+14%	+18%

The parameter values utilized for these simulations are those shown in Table 2. Work intensity was set at an A_UT_ of 78.2 in simulations in the untrained state and in the trained state for the same absolute work intensity, and at an A_UT_ of 89.3 in simulations of the trained state for the same relative work intensity, respectively.

no st.-sign., no statistically significant difference.

The relative changes in *t*_0.63_ and $\tau_{{\dot{V}}_{O2}}$ for the three cases of training for the same absolute and relative changes in power output are presented in Table 2. In general, the results of the simulations agree well with the experimental data. For example, $\tau_{{\dot{V}}_{O2}}$ increased by 15%–19% in simulations, closely matching the increase in the experiment (16%). The $\tau_{{\dot{V}}_{O2}}$ for the same absolute power output decreased by 20%–27% in the simulations, compared with a 38% reduction in the experiment. For the same relative exercise intensity, the $\tau_{{\dot{V}}_{O2}}$ increased by 10%–15% in simulations, while the experimental data displayed a similar trend (Fig. 2D). The training-induced shortening of *t*_0.63_ by 11%–13% in simulations compared with 19% in the experiment, whereas CP increased by 14%–19% in the simulations versus 14% in the experiment. Finally, $\tau_{{\dot{V}}_{O2}}$ increased across all simulated training cases, with trained 3 (+18%) aligning most closely with the experimental value of +21%.

### Simulated Pretraining and Posttraining A_UTcrit_ (PO)-*t*_end_ (Duration) Dependence

The simulated power-duration (PO-*t*_end_) relation was hyperbolic and the power−1/duration (PO−1/t_end_) relation was linear (Fig. 6). CP (A_UTcrit_) increased as a result of training, from an A_UT_ of 75.8 in the untrained state to 86.5 (trained 1), 89.5 (trained 2), or 88.8 (trained 3). $\tau_{{\dot{V}}_{O2}}$ ([A_UT_−A_UTcrit_] × *t*_end_) increased as result of training, from 50.4 in the untrained state to 53.5 (trained 1), 57.6 (trained 2), or 59.2 (trained 3).

The relative changes in A_UTcrit_ (CP) and (A_UT_−A_UTcrit_) × *t*_end_ ($\tau_{{\dot{V}}_{O2}}$) for the three cases of training are presented in Table 2. Trained 1 was the situation that most closely matched the experimental increase in CP (both +14%), whereas trained 3 most closely matched the experimentally-induced increase in $\tau_{{\dot{V}}_{O2}}$ (experiment: +21%, simulation: +18%).

## DISCUSSION

The main findings of this study were that 1) $\dot{V}O_{2}$ and CP were strongly inversely correlated both before and after 2-wk of exercise training, and 2) the intervention-induced changes in $\tau_{{\dot{V}}_{O2}}$ and CP were also associated with one another. These findings are consistent with our hypotheses, and the notion that $W^{\prime }$and CP share common determinants rooted in the bioenergetics of muscular exercise. Computer simulations suggested that the main factor responsible for these phenomena was the training-induced increase in OXPHOS activity (k_OX_). Inconsistent with our hypotheses, however, was that 3) $\dot{V}O_{2sc}$ was not related to the amplitude of the $\dot{V}O_{2sc}$ before or after the 2 wk of exercise training. A training-induced increase in $W^{\prime }$ was accompanied by a reduced $\dot{V}O_{2sc}$ amplitude at the same absolute power output, whereas at the same relative exercise intensity posttraining, the amplitude of the $\dot{V}O_{2sc}$ was unchanged. As such, and also inconsistent with our hypotheses, the intervention-induced changes in $W^{\prime }$ and the $\dot{V}O_{2}$ at both the same absolute and relative power outputs were not correlated. These findings argue against a single straightforward mechanistic association between $\dot{V}O_{2sc}$and the $W^{\prime }$ in the present study and suggest instead that the determinants of this relation are multifactorial. Computer simulations suggest that the factors responsible for the training-induced changes in the $W^{\prime }$ and $\dot{V}O_{2sc}$ were likely related to an increase in OXPHOS activity (k_OX_), a decrease in the accessible phosphate (+ creatine) pool (P_acc_), and/or an increase in Pi_peak_.

### $W^{\prime }$–CP Relation

The strength and consistency of the relation between $W^{\prime }$ and CP (9,15,48) has lent strength to the notion that these two variables share common bioenergetic determinants. Experimental studies designed to test the hypothesis of a mechanistic linkage between these variables demonstrated that interventions designed to experimentally speed the $\dot{V}O_{2sc}$ kinetics such as priming exercise (18,19) or hyperoxia (20) increased CP, whereas interventions designed to experimentally slow the $\dot{V}O_{2sc}$ kinetics, such as the initiation of exercise from an elevated baseline power output (21, 22) decreased CP. However, in most of these studies (see (20)), the intervention-induced changes in $\dot{V}O_{2sc}$ and CP were not correlated. Additionally, it is likely that various biochemical factors contribute to differing extents to the variability in $\dot{V}O_{2sc}$ and CP values observed both between individuals (interindividual variability) and across different interventions (e.g., training, hypoxia, priming exercise, or elevated baseline power output). In the present study, however, we found a strong, inverse relation between $W^{\prime }$and CP both before and after training (Fig. 3A, B). Moreover, the intervention-induced changes in $W^{\prime }$and CP were correlated with one another (Fig. 3C), implying that these variables move in close proportion after specific training. These findings are broadly consistent with computer simulations of the human bioenergetic system, where changes in OXPHOS activity, ESA intensity, Pi_crit_, Pi_peak_, and intramyocellular O_2_ pressure change $\dot{V}O_{2}$and CP in opposite directions (34,38). The increase in OXPHOS activity, which is known to occur with exercise training (49–53), reduces the degree of intramuscular metabolic perturbation incurred for a given power output (34,53). Hence, the findings of the present study are consistent with the notion that $\dot{V}O_{2sc}$and CP share common bioenergetic determinants related to the capacity to sustain high-intensity exercise (15,17,35). In particular, and consistent with recent reports (39), the simulations in the present study indicated that the main factor responsible for the training-induced changes in CP and $W^{\prime }$was k_OX_ (i.e., the rate constant for OXPHOS activity). This is consistent with recent work (39), which proposed that the inverse relation between CP and *t*_0.63_ ($W^{\prime }$) relation (9) arises from interindividual variability in OXPHOS activity (k_OX_) and possibly, to a lesser extent, ESA intensity (A_OXmax_) and the accessible phosphate pool (P_acc_).

The relation between k_OX_, CP, and $\dot{V}O_{2sc}$is likely driven through the moderating role that k_OX_ plays in determining metabolite changes (i.e., ΔP_i_, ΔPCr, Δfree ADP, etc.) during work transitions. For example, an increase in k_OX_ abates the rise in P_i_ and free ADP and fall in PCr during the transition from rest to work (34,38), and a smaller magnitude of metabolite changes at the onset of exercise in the trained state (i.e., reduced ΔP_i_, ΔPCr, Δfree ADP compared with the untrained state) results in lower values of $\dot{V}O_{2}$and thus more rapid $W^{\prime }$ kinetics (54). Recent simulation studies involving the P_i_ double-threshold mechanism suggest that such smaller metabolite (especially P_i_) changes posttraining result in an increase in CP. This occurs because when exercising at a power output that was above CP before training, P_i_ no longer reaches Pi_peak_ ((34), and related discussion therein). Hence, the likely mechanism explaining training-induced changes in CP and $\dot{V}O_{2sc}$ in the present study is a reduction in metabolite changes incurred during the transition from rest to work, consequent to an increase in k_OX_. The increase in k_OX_ is, in turn, likely a result of increases in mitochondrial volume density (55), cristae surface area density (56) or the activity of OXPHOS complexes (52), which are known to vary with training status.

### $\dot{V}O_{2sc}$–$\dot{V}O_{2max}$ Relation

It was previously demonstrated that the amplitude of the $W^{\prime }$ was positively correlated with $W^{\prime }$(17). This relation was interpreted to reflect the fact that the $\dot{V}O_{2sc}$ reflects a loss of skeletal muscle efficiency (57,58) and the development of muscle fatigue (27,59), the limits of which define exercise intolerance during exercise above CP. However, a decrease in muscle efficiency *per se* should decrease the amount of work performed above CP. It was recently proposed that the interindividual variability in the $\dot{V}O_{2sc}$–$W^{\prime }$ relation was largely due to variability in k_add_ (the rate constant for additional ATP usage underlying the slow component of $\dot{V}O_{2sc}$ and metabolites) (39). In this previous study, increases in k_add_ alone elevated both $W^{\prime }$ and $\dot{V}O_{2sc}$ (39). It may seem paradoxical that an increased k_add_ elevates $\dot{V}O_{2sc}$, as an elevated k_add_ accelerates the attainment of Pi_peak_ at the same absolute work intensity, which tends to diminish the work performed above CP. However, it also decreases CP itself, which tends to increase the work performed above CP (39). Nevertheless, the results of the present study argue against a straightforward positive correlation between the amplitude of the $W^{\prime }$ and $W^{\prime }$. $\dot{V}O_{2sc}$ was increased by our training intervention, whereas the amplitude of the $\dot{V}O_{2sc}$ was reduced at the same absolute power output posttraining. However, because this power output resided below the posttraining CP, we decided to conduct additional trials performed at the same relative intensity as preintervention. Therefore, a major strength of the present study was the comparison of the amplitude of the $W^{\prime }$ at the same absolute and relative intensities pre- and posttraining. When performing comparisons at the same relative exercise intensities pre- and posttraining, however, the amplitude of the $\dot{V}O_{2sc}$ was unaltered, and their training-induced changes were also not correlated. Consistent with these findings, previous studies have demonstrated that endurance training typically blunts the $W^{\prime }$ amplitude at a fixed absolute power output (43), whereas $W^{\prime }$ is typically unchanged (41). Hence, these data clearly demonstrate that the relation between $\dot{V}O_{2sc}$ and the $\dot{V}O_{2sc}$ slow component is not immutable, but can be modified under specific physiological conditions.

The results of the present study, along with those of prior experimental work, argue that there is no simple, unitary relation between the $W^{\prime }$ and $W^{\prime }$, and show that these variables can change in opposite directions under certain circumstances. For example, the initiation of exercise from an elevated baseline power output in the supine position caused $W^{\prime }$ to increase, whereas the amplitude of the $W^{\prime }$ was reduced (22). Hyperoxic gas inspiration, on the other hand, reduces the rate of development of the $W^{\prime }$ and intramuscular metabolite slow components and reduces $W^{\prime }$ (31,40). However, when the $W^{\prime }$ amplitude is determined at the limit of tolerance in hyperoxia, the $W^{\prime }$ is increased due to the increased $W^{\prime }$, whereas $\dot{V}O_{2sc}$ is reduced (40). Similarly, the performance of prior heavy exercise has been shown to increase $W^{\prime }$ alongside a reduction in the amplitude of the $\tau_{{\dot{V}}_{O2}}$ (60–62). Hence, it is clear that under certain circumstances, the $W^{\prime }$ and $\dot{V}O_{2}$ can be dissociated from one another. The original results of Murgatroyd et al. (17), which demonstrated a strong positive correlation between the $\dot{V}O_{2}$ and $\tau_{{\dot{V}}_{O2}}$ were determined under conditions in which the tolerable duration of exercise was strictly controlled (i.e., 6 min therein). Hence, as within the severe domain the magnitude of the $\dot{V}O_{2}$ depends strongly on the tolerable duration of exercise, the results of that study are likely specific to the conditions studied therein. In the present study, the primary comparisons were performed at 10% above the pre- and postintervention CP. While this attempt to control for relative exercise intensity resulted in all subjects exercising within the same intensity domain, the tolerable duration was not uniform across subjects (range 649–1241 s). Hence, it remains to be determined whether an experimental dissociation between the amplitude of the $\dot{V}O_{2max}$ and $W^{\prime }$ would be observed with training if the tolerable duration of exercise was controlled for.

Given the discrepancies between our results and those of Murgatroyd et al. (17), we aimed to explore the biochemical factors which could produce an elevation in $\dot{V}O_{2sc}$alongside the training-induced reduction of $\dot{V}O_{2sc}$ via computer modeling. The results of the computer simulations suggested that different parameters determine the $W^{\prime }$ and $\dot{V}O_{2max}$ (i.e., changes in OXPHOS activity, the accessible phosphate (+creatine) pool, and/or increases in Pi_peak_), pointing towards a multifactorial determination of the $\dot{V}O_{2sc}$–$W^{\prime }$ relation. Among the simulations performed, trained 3, which involved a simultaneous increase in k_OX_, and Pi_peak_, produced the increase in $\dot{V}O_{2sc}$ which most closely matched the experimental findings (i.e., +18% and +21%, respectively). On the other hand, trained 2 (involving an increase in k_OX_ and decrease in P_acc_) produced the changes in the $\dot{V}O_{2sc}$ that most closely matched the experimental findings (i.e., −27% and −38% for the same absolute work rate and +15% and +10%–15% for the same relative work rate, respectively). Overall, these findings suggest that the synergistic effects of increases in k_OX_, decreases in P_acc_, and potential increases in Pi_peak_ seem to best account for the present experimental data. One explanation for the discrepancy between the present and previous studies (17), therefore, is that under conditions where the tolerable duration of exercise is strictly controlled, the $W^{\prime }$–$W^{\prime }$ relation is likely determined by a single factor (k_add_). Conversely, under other conditions, multiple factors (e.g., k_add_, P_acc_, k_OX_, and Pi_peak_) can contribute to this relation, leading to a more complex interplay. However, the lack of control of exercise duration in our study cannot explain the fact that exercise training led to an increase in $W^{\prime }$, whereas most previous studies observed a decrease or no change in $\dot{V}O_{2sc}$ (41–43). Therefore, an alternative explanation for these discrepancies could be differences in the characteristics of study participants or nature of the interventions employed across studies. Indeed, the relatively intense nature of the intervention used herein, combined with the low baseline training status of the participants, may partly explain the training-induced increase in $\tau_{{\dot{V}}_{O2}}$. It should be stressed that in previous computer simulations concerning the training-induced changes in $\dot{V}O_{2sc}$, a decrease in Pi_peak_ was applied to varying degrees, leading to a decrease or negligible change in $\tau_{{\dot{V}}_{O2}}$ (see e.g., (34)) whereas in trained 3 in the present study, Pi_peak_ was increased, resulting in a substantial increase in $\tau_{{\dot{V}}_{O2}}$. Therefore, it is possible that changes in Pi_peak_ may be the primary factor responsible for the training-induced changes in $\dot{V}O_{2sc}$ (although not necessarily for the interindividual variability in $W^{\prime }$, where k_add_ seems to be crucial). Thus, interventions that alter the balance of these determinants may obscure the $W^{\prime }$–$\dot{V}O_{2sc}$ relation observed in previous studies.

### Whole-Body Systemic Properties

In the present study, we primarily considered the biochemical system properties of skeletal muscle in relation to the CP–$\dot{V}O_{2sc}$ and $W^{\prime }$–$W^{\prime }$ relations. This is justified, as many system properties at the level of whole-body, integrative physiology are rooted in intramuscular bioenergetics. Nevertheless, it is well-known that the power-duration relation parameters and the $W^{\prime }$ kinetics can be influenced by whole-body systemic properties such as rates of convective and diffusive O_2_ delivery. However, it is typically agreed that in young healthy physically active individuals exercising in the upright position, O_2_ supply limitations are small and do not appreciably impact system properties (the “tipping point” hypothesis (63), later modified to the “smooth transition” idea by Korzeniewski (38)). However, our subjects were sedentary, as evinced by their relatively large baseline $\dot{V}O_{2}$, and therefore O_2_ delivery-limited $\dot{V}O_{2}$ kinetics at baseline are quite likely in these individuals. On the other hand, potential limitations in O_2_ supply can only act on cell energetics through a lowered cellular O_2_ concentration. O_2_ is the substrate for cytochrome oxidase, therefore a lower intramyocyte O_2_ concentration directly decreases the “effective” activity (k_OX_) of OXPHOS. In this way, the whole-body systemic limitations ultimately manifest at the biochemical level of skeletal muscle. Related theoretical frameworks have proposed that intracellular O_2_ distribution may also be influenced by myoglobin-mediated nitric oxide signaling and local modulation of cytochrome oxidase activity (64–66), but these mechanisms were not examined in the present study.

### Model Parameters versus System Variables

A systematic analysis of the impact of several system parameters (k_OX_, A_OXmax_, Pi_peak_, Pi_crit_, k_add_, P_acc_, and O_2_ concentration) on system variables such as $\tau_{{\dot{V}}_{O2}}$, CP, *t*_0.63_, $\dot{V}O_{2}$, $\tau_{{\dot{V}}_{O2}}$, and *t*_term_ (time to exercise termination) was performed in detail in previous work (34,35,37–39,67). These studies demonstrate the sensitivity of model behavior to individual parameter changes across physiologically plausible ranges.

In the present study, the simulations were therefore not intended to repeat an exhaustive parameter sensitivity analysis, but rather to examine whether experimentally observed training-induced changes could be reproduced within this established modeling framework using physiologically plausible parameter adjustments. Accordingly, several tens of parameter combinations were explored to assess model behavior, and representative cases relevant to the present experimental findings are presented.

### Limitations

One of the limitations of this study is that, although we conducted a comparison of the $\dot{V}O_{2}$ with $\tau_{{\dot{V}}_{O2}}$ at the same absolute and relative intensity posttraining (i.e., 10% above the new CP), we did not control for the tolerable duration of exercise at either time point. During severe-intensity exercise, the development of the slow component is truncated by $\tau_{{\dot{V}}_{O2}}$. Therefore, we decided to compare the pre- and posttraining $\tau_{\dot{V}}$ amplitude at the same absolute and relative work intensities at a fixed duration after the onset of exercise. Indeed, exercise duration tended to be slightly lower during exercise 10% above CP posttraining (although not significantly, *P* = 0.19), with a large degree of between-subject variability in the change in time to the limit of tolerance. Hence, the apparent lack of a relation between training-induced changes in $\tau_{{\dot{V}}_{O2}}$ and the $\tau_{{\dot{V}}_{O2}}$ may be partly explained by the variable change in tolerance time from pre- to postintervention between subjects at 10% above CP. However, the lack of relation between these variables at the same absolute power output argues against this possibility and suggests instead that no simple positive relation between the $\tau_{{\dot{V}}_{O2}}$ and $\dot{V}O_{2}$ was present in these participants/conditions. Additionally, the lack of control over exercise duration does not explain the training-induced increase in $W^{\prime }$ observed in our study, whereas other studies, including the seminal work of Gaesser and Wilson (41), have reported either a decrease or no change (43,43). Therefore, differences in participant characteristics and the nature of the training intervention may provide an alternative explanation. Future research should explore the relation between training-induced alterations $\dot{V}O_{2}$ and the $W^{\prime }$ while rigorously controlling for the tolerable duration of exercise, as per Murgatroyd et al. (17), to further probe the association between these parameters.

The dynamic computer model used in this study, like all such models, represents only a simplified approximation of the complex physiological system. The “standard” version of the computer dynamic model used in this study, based on several hundreds of experimental studies, represents typical young, healthy, untrained but physically active individuals exercising in the upright position. As such, it predicts average or typical (most frequent) values for variables such as *t*_0.63_ (24 s) or CP (>200 W) (15), which differ from the experimental baseline values of $\dot{V}O_{2}$ (47 s) and CP (155 W) observed in the present study and reflect values more typical of sedentary individuals. For instance, the simulated value of *t*_0.63_ is close to the mean values reported across a wide range of experimental studies (see Poole and Jones (68) for a review). While re-parameterization could, in principle, shift absolute kinetic values, the tightly coupled nature of the model means that matching the present experimental data would require coordinated changes across multiple interdependent parameters rather than adjustment of a single parameter. Given that the model was developed to capture general system behavior across diverse experimental conditions, it was not retuned to reproduce cohort-specific variability, and the simulations are therefore intended to illustrate mechanistic relations rather than to explain differences between individual experimental datasets. Therefore, the comparison focuses on relative training-induced changes rather than absolute values. Despite these limitations, however, the general conclusions derived from the simulations remain robust, and the relative changes induced by the experimental intervention are closely reflected by the trained simulations employed herein. Overall, the simulations aligned well with the experimental data, capturing the most important trends and providing insight into the mechanistic basis of training-induced changes. These results highlight the utility of the model in predicting biochemical adaptations to exercise training.

## CONCLUSIONS

The results of the present study are consistent with the notion that fast $W^{\prime }$ kinetics are the consequence of adaptations that enable the ability to meet the energetic demands of exercise in a dynamic equilibrium. Specifically, both before and after a short-term, high-intensity training programme, $\dot{V}O_{2}$was strongly inversely correlated with CP, and the training-induced changes in both parameters were also correlated. These findings provide further evidence that the mechanisms determining the rate of adaptation of O_2_ consumption at the onset of exercise are similar to those determining the ability to sustain exercise in a metabolic steady-state. Computer simulations suggest that the main factor responsible for the training-induced changes in *t*_0.63_ ($W^{\prime }$) and CP is an increase in the rate constant describing OXPHOS activity, k_OX_. The amplitude of the $\dot{V}O_{2}$ was not associated with $\dot{V}O_{2}$ either before or after training. $\dot{V}O_{2}$ was increased as a result of training, whereas the amplitude of the $W^{\prime }$ was reduced posttraining at the same absolute power output and unaffected at the same relative work intensity. The training-induced changes in the $\dot{V}O_{2max}$ and $\dot{V}O_{2max}$ were not correlated. Hence, these findings indicate a training-induced dissociation between these parameters. While it remains likely that $\dot{V}O_{2max}$ and the slow component shares similar mechanistic determinants, the present study highlights that this relation is not a straightforward association, but is instead dependent upon multiple factors under different conditions. Computer simulations suggest that in our study, these factors may comprise OXPHOS activity, the accessible phosphate (+creatine) pool, and/or peak P_i_, at which exercise is terminated because of fatigue.

T. H. L. was supported by Hubei Province Grant (B2023136). R. P. G. was supported by Nederlandse Organisatie voor Wetenschappelijk Onderzoek Award Number: https://doi.org/10.61686/CTDKS25250 and De Nederlandse Organisatie voor Gezondheidsonderzoek en Zorginnovatie Award Number: 10091012410013.

The authors thank the participants for their efforts during this study. No conflicts of interest were disclosed. T. H. L. and R. P. G. contributed to conceptualization and design. T. H. L. and I. L. W. was responsible for data collection. T. H. L., R. P. G., L. V., I. L. W., S. K., S. M., and B. K. were responsible for data analysis, interpretation, and drafting of the article. All authors reviewed the article and provided critical feedback. All authors approved the final manuscript. The results of this study are presented clearly, honestly, and without fabrication, falsification, or inappropriate data manipulation. The results of the present study do not constitute endorsement by the American College of Sports Medicine. The data are available upon request.
